# Residual Postural-Control Differences After Anterior Cruciate Ligament Reconstruction: Dual-Task Postural Control and Associations with Time Since Return to Sport

**DOI:** 10.3390/jcm15218699

**Published:** 2026-11-07

**Authors:** Shaker Hassan S. Alshehri, Nizar Algarni, Batool Abdulelah Alkhamis, Ghada Mohamed Koura, Ahmed Mohamed Fathi Elshiwi, Saleh M. Kardm, Amal Awad Saeed Alqahtani, Ravi Shankar Reddy, Ajay Prashad Gautam, Zeinab A. Ali, Maram Y. Al Ahmari

**Affiliations:** 1 Department of Orthopedic Surgery, College of Medicine, King Khalid University, Abha 61421, Saudi Arabia; shhshehri@kku.edu.sa; 2 Department of Orthopedic Surgery, College of medicine, King Saud University, Riyadh 11461, Saudi Arabia; 3 Program of Physical Therapy, Department of Medical Rehabilitation Sciences, College of Applied Medical Sciences, King Khalid University, Abha 61423, Saudi Arabiagibrahim@kku.edu.sa (G.M.K.); rshankar@kku.edu.sa (R.S.R.); 4 Physical Therapy for Musculoskeletal Disorders and Its Surgery, Faculty of Physical Therapy, Cairo University, Giza 12613, Egypt; 5 Department of Surgery, College of Medicine, Najran University, Najran 11001, Saudi Arabia; 6 Department of Physical Therapy for Surgery, Faculty of Physical Therapy, Cairo University, Giza 12613, Egypt; 7 Department of Physical Therapy and Health Rehabilitation, College of Applied Medical Science, Jouf University, Sakaka 72388, Saudi Arabia; 8 Department of Physical Therapy, Prince Sultan Military Medical City, Riyadh 11159, Saudi Arabia

**Keywords:** anterior cruciate ligament reconstruction, postural balance, proprioception, cognition, return to sport, rehabilitation

## Abstract

**Background/Objectives:** Residual sensorimotor deficits may persist after anterior cruciate ligament reconstruction (ACLR), even after clearance for unrestricted sport. This study compared unilateral postural control between individuals after ACLR and healthy controls under visual deprivation and cognitive dual-task conditions and examined associations between dual-task cost and time since reconstruction and return to activity. **Methods:** This cross-sectional study included 35 physically active adults 9–24 months after primary unilateral ACLR and 35 age-, sex-, body mass index-, and activity-matched controls. Force-platform posturography measured center-of-pressure (COP) path length, ellipse area, and mean velocity during unilateral stance with eyes open, eyes closed, and concurrent serial subtraction. COP path-length dual-task cost (DTC) was compared between groups and limbs. Correlation and multiple linear regression analyses examined associations with postoperative and return-to-activity duration. **Results:** Eyes-closed COP path length was greater in reconstructed limbs than in control reference limbs (720.00 ± 118.00 versus 552.00 ± 92.00 mm; *p* < 0.001). Reconstructed-limb DTC exceeded control reference-limb DTC (17.45 ± 10.20% versus 6.97 ± 6.80%; *p* < 0.001; Cohen’s d = 1.21) and contralateral-limb DTC (11.00 ± 8.40%; *p* < 0.001). Longer time since ACLR (r = −0.38; *p* = 0.024) and return to unrestricted activity (r = −0.44; *p* = 0.008) were associated with lower DTC. Both associations remained significant in a two-predictor regression model (adjusted R^2^ = 0.20). **Conclusions:** Postural-control differences persisted after ACLR despite clearance for unrestricted activity and were more evident under visual deprivation and cognitive loading. These cross-sectional associations support further investigation of dual-task posturography in postoperative assessment. The clinical significance and predictive value of these static posturographic measures for return-to-sport readiness and subsequent injury risk remain to be established.

## 1. Introduction

Anterior cruciate ligament (ACL) injury is a clinically important knee injury among physically active individuals and frequently requires ACL reconstruction (ACLR) to restore mechanical stability and facilitate return to sport [1]. However, successful surgical reconstruction does not necessarily indicate complete restoration of sensorimotor function [2]. The ACL contributes to neuromuscular regulation through mechanoreceptive afferent information involved in proprioception, joint-position awareness, and postural control [3]. Injury and subsequent reconstruction can therefore be accompanied by persistent alterations in sensory integration and motor control [3]. These deficits are clinically relevant because return to sport exposes individuals to situations requiring rapid integration of visual, proprioceptive, and cognitive information while maintaining lower-extremity stability [3]. Consequently, assessment strategies that challenge the sensorimotor system may provide information not apparent during conventional clinical evaluation. This distinction between restoration of mechanical knee stability and recovery of functional postural control provides a rationale for examining postoperative performance under conditions that challenge sensory integration and attentional resources [3]. Visual deprivation increases reliance on nonvisual sensory information, whereas concurrent cognitive tasks introduce competing attentional demands that may influence postural regulation. Incorporating these conditions into force-platform assessment may therefore help characterize residual postural-control differences that are less apparent during uncomplicated eyes-open stance [4]. However, such measurements should be considered complementary to established clinical and functional assessments, as their ability to inform return-to-sport readiness or predict subsequent injury has not been established.

Postural-control abnormalities after ACL injury and reconstruction have been demonstrated using center-of-pressure (COP)-based measures during force-platform testing [5]. Liu et al. [6] reported altered postural control following ACLR and highlighted the contribution of proprioceptive dysfunction to postoperative sensorimotor performance. Attalin et al. [7] similarly demonstrated impaired postural control associated with ACL insufficiency, supporting the importance of ligament-related afferent information for postural regulation. A systematic review by Gholami et al. [8] further indicated that measurable postural-control deficits may persist following ACLR. Visual availability is particularly relevant because removal of vision increases dependence on somatosensory information, potentially exposing deficits that are less evident during eyes-open testing [8]. In parallel, dual-task paradigms impose competing attentional demands on postural regulation. Sharouf et al. [9] demonstrated altered postural control during cognitive dual-tasking in individuals with unilateral ACL injury, supporting the relevance of cognitive-motor interference when evaluating ACL-related sensorimotor function.

Despite this evidence, important aspects of postoperative assessment remain insufficiently characterized. Many postural-control studies evaluate visual or cognitive challenges separately, limiting interpretation of how residual deficits manifest across distinct sensory and attentional demands within the same clinically recovered population [10]. Furthermore, group-level comparisons alone may obscure reconstructed-to-contralateral limb asymmetry, while absolute dual-task performance does not quantify the proportional deterioration attributable to cognitive loading [10]. Evaluating COP outcomes across visual conditions together with COP path-length dual-task cost (DTC) may therefore provide a more specific characterization of residual sensorimotor impairment [11]. Determining whether DTC is associated with time since reconstruction and time since return to unrestricted activity is also clinically relevant for interpreting postural performance across the postoperative period [11]. Although previous studies have established that postural-control alterations may persist following ACLR and have independently examined the effects of visual deprivation or cognitive loading [7,8,9,10,11], less is known about how these responses coexist within the same postoperative population after clearance for unrestricted activity. In particular, the combined characterization of visual-deprivation responses, cognitive dual-task cost, reconstructed-to-contralateral limb differences, and their associations with time since reconstruction and return to unrestricted sport or activity remains insufficiently investigated. The present study addresses this gap by integrating these complementary assessments within a single cohort of physically active adults following primary unilateral ACLR and comparing their postural performance with that of matched healthy controls. This approach provides a more comprehensive characterization of residual postural-control differences across sensory and attentional conditions while examining their relationships with postoperative and return-to-activity duration.

Accordingly, this study aimed to determine whether physically active adults following primary unilateral ACLR demonstrated persistent static postural-control deficits compared with matched healthy controls, with particular emphasis on reconstructed-limb performance and the response to visual deprivation. A second objective was to determine whether cognitive dual-tasking disproportionately impaired reconstructed-limb postural control, whether COP path-length DTC differed between limbs, and whether reconstructed-limb DTC was associated with postoperative and return-to-activity duration. We hypothesized that ACLR participants would demonstrate greater COP path length, ellipse area, and mean COP velocity than healthy controls, with larger deficits under eyes-closed conditions; that the reconstructed limb would demonstrate greater DTC than the contralateral limb and the control reference limb; and that longer time since ACL reconstruction and longer time since return to unrestricted activity would be associated with lower reconstructed-limb DTC.

## 2. Materials and Methods

### 2.1. Study Design, Ethics, and Settings

This cross-sectional study was conducted between February 2025 and January 2026 at the Orthopedic and Rehabilitation Clinic, Department of Physical Therapy, King Khalid University, Kingdom of Saudi Arabia, and included physically active adults following primary unilateral anterior cruciate ligament reconstruction and matched healthy controls. Ethical approval was obtained from the Institutional Review Board of King Khalid University (approval no. [KKU-163-2025-31]), and written informed consent was obtained from all participants before study procedures were initiated. The study was conducted in accordance with the principles outlined in the Declaration of Helsinki.

### 2.2. Sample Size Calculation

An a priori sample-size calculation was performed using G*Power version 3.1.9.7 (Heinrich Heine University Düsseldorf, Düsseldorf, Germany) for the primary between-group comparison of reconstructed-limb COP path length between ACLR participants and healthy controls [12]. Assuming a standardized between-group effect size of Cohen’s d = 0.68, a two-sided α level of 0.05, statistical power of 80%, and equal group allocation, 35 participants per group were required. The target sample was therefore 70 participants, comprising 35 participants following primary unilateral ACLR and 35 healthy controls.

### 2.3. Participants

Participants were recruited from physically active adults attending the Orthopedic and Rehabilitation Clinic and through clinician referral and local recruitment advertisements. The ACLR group comprised individuals with a documented history of primary unilateral anterior cruciate ligament reconstruction, verified from available surgical/clinical records and confirmed during screening through medical history and clinical examination. Healthy controls were recruited from the same general physically active population and were frequency matched to the ACLR group for age, sex, body mass index, and habitual physical activity level. Matching was performed at the group level rather than as fixed participant pairs; therefore, observations from the ACLR and control groups were treated as independent in between-group statistical analyses. Before enrollment, all candidates underwent standardized eligibility screening that included demographic and activity history, lower-extremity injury and surgical history, current musculoskeletal symptoms, and relevant neurological and vestibular history.

Participants were eligible for the ACLR group if they were physically active adults aged 18–35 years, had undergone primary unilateral ACL reconstruction 9–24 months previously, had completed postoperative rehabilitation, and had received medical clearance for unrestricted sport or physical activity. Unrestricted return to sport/activity was defined as documented clearance to resume sport or habitual physical activity without postoperative activity restrictions. The date of clearance was obtained from the participant’s clinical or rehabilitation record and verified during the baseline interview. Time since return to unrestricted sport/activity was calculated in months from the documented clearance date to the date of posturographic assessment. Return to unrestricted sport/activity was operationally defined by documented medical clearance rather than verified restoration of preinjury sporting participation. Although participants had completed postoperative rehabilitation and were physically active at assessment, their return to preinjury sporting level, competitive intensity, and performance was not independently established. Accordingly, time since return to unrestricted sport/activity represents the interval since medical clearance and should not be interpreted as the duration of participation at the preinjury sporting level. Individuals were excluded if they had undergone revision ACL reconstruction, had a history of contralateral ACL injury or reconstruction, had undergone major concomitant ligament reconstruction, had a recent lower-extremity musculoskeletal injury capable of affecting postural performance, or had a clinically relevant neurological or vestibular disorder. Healthy controls were required to have no history of ACL injury or reconstruction and no current lower-extremity condition affecting balance or participation in physical activity; the same neurological, vestibular, and recent musculoskeletal-injury exclusions were applied. Eligibility was confirmed before posturographic testing, and baseline assessment documented age, sex, height, body mass, body mass index, limb dominance, and weekly sport/training exposure. For the ACLR group, operated limb, graft type, concomitant meniscal treatment, time from injury to reconstruction, time since reconstruction, time since return to unrestricted sport/activity, supervised rehabilitation duration, and postoperative knee range of motion were additionally recorded.

### 2.4. Posturographic Testing Procedures

**Figure 1:**
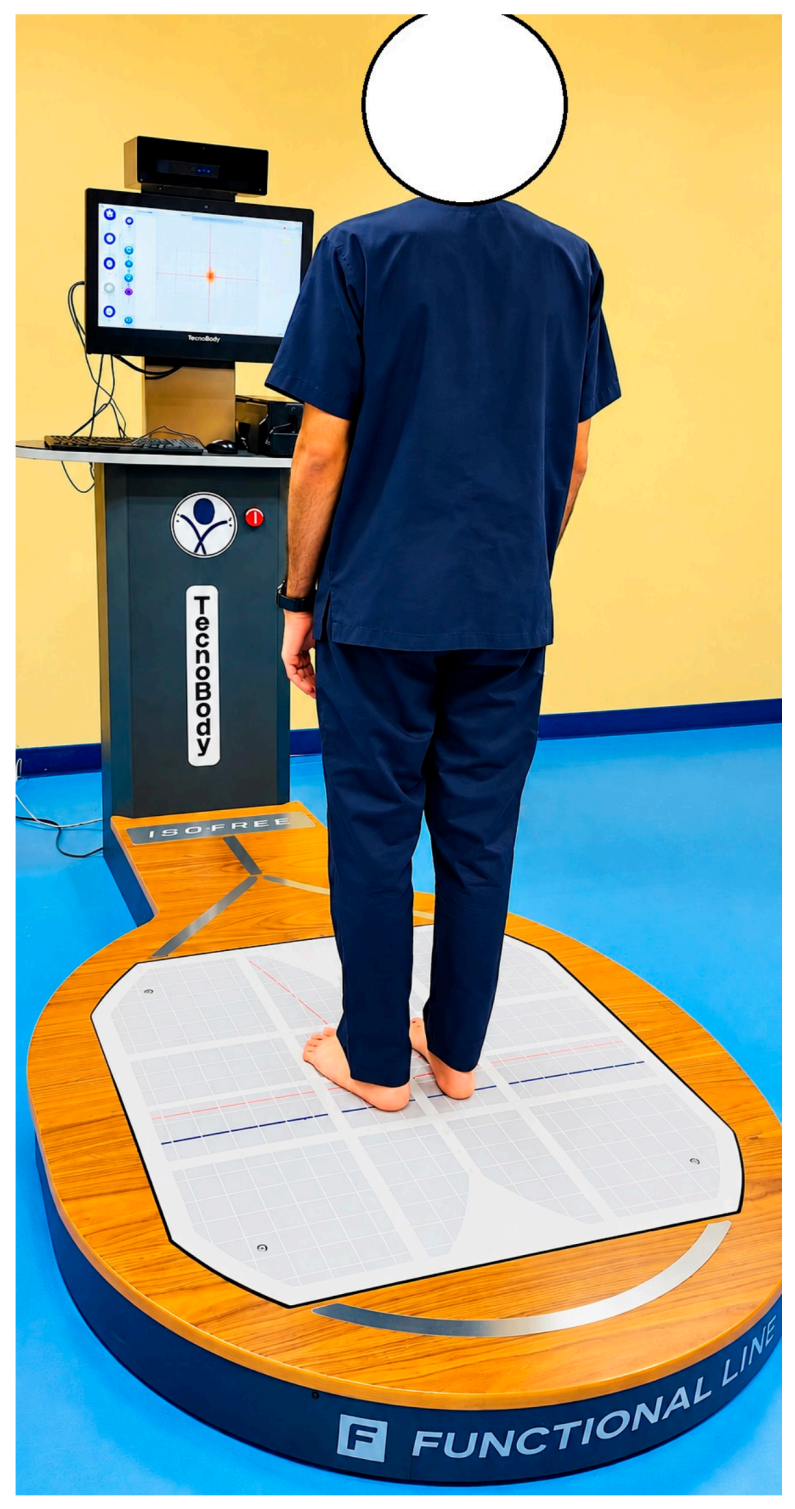
TecnoBody ISO-Free force-platform system used to assess unilateral static postural control under standardized testing conditions.

Postural-control testing was performed using the Technobody Isofree posturographic force-platform system (TecnoBody S.p.A., Dalmine, Bergamo, Italy) in a quiet, well-lit clinical laboratory under standardized environmental conditions (Figure 1) [13].

The force platform was calibrated before each testing session according to the manufacturer’s procedures, and COP signals were acquired at 100 Hz [13]. Participants performed testing barefoot to minimize variability attributable to footwear. Before data collection, each participant received standardized instructions and completed one familiarization trial for each experimental condition. During testing, participants stood unilaterally near the center of the platform with the supporting foot oriented forward, the hands placed on the iliac crests, and the non-supporting limb flexed at the knee sufficiently to prevent contact with the platform or stance limb. Participants were instructed to maintain the prescribed position and minimize unnecessary trunk and limb movements throughout each trial. Each recorded trial lasted 30 s. Three valid trials were obtained for each limb and experimental condition, with 60 s of seated rest between trials, and the mean of the three valid trials was retained for analysis. The order of tested limb and experimental condition was randomized using a computer-generated sequence to minimize systematic learning, fatigue, and order effects. A trial was considered invalid and repeated when the participant moved the stance foot from its initial position, contacted the platform or floor with the non-supporting foot, used external support, removed the hands from the iliac crests, opened the eyes during an eyes-closed trial, or was unable to complete the full 30-s recording. Invalid trials were repeated to obtain three recordings that satisfied the standardized testing criteria for each limb and experimental condition. Only valid trials contributed to the participant-level posturographic outcomes. The number and frequency of unsuccessful attempts were not included as separate study outcomes; therefore, their potential functional significance and associations with postural-control performance could not be evaluated. Trial invalidation was treated as a data-quality criterion rather than an independently validated indicator of functional instability. COP path length, ellipse area, and mean COP velocity were obtained from the Technobody software using the same acquisition and processing settings for all participants and conditions [14].

### 2.5. Center-of-Pressure Path Length

COP path length was designated a priori as the primary posturographic outcome and was expressed in millimeters [6]. It represented the cumulative distance traveled by the COP trajectory over the complete 30-s recording period and was obtained from the Technobody Isofree software for each valid trial [14]. The mean of three valid trials for each limb and condition was used as the participant-level value. Larger values indicated greater postural sway. COP path length was analyzed in its original continuous scale, without transformation or application of a diagnostic cutoff. The primary between-group comparison involved the ACLR reconstructed limb and the control reference limb, with eyes-closed testing representing the principal sensory-challenge condition.

### 2.6. COP Ellipse Area

COP ellipse area was a secondary posturographic outcome and quantified the spatial dispersion of the COP trajectory during the 30-s unilateral stance trial [5]. Values were calculated automatically by the Technobody Isofree software and expressed in square millimeters (mm^2^) [5]. The same software-derived definition and processing settings were applied to every participant, limb, and experimental condition. The mean of three valid trials was retained for analysis. Larger ellipse-area values represented a greater spatial distribution of COP displacement and therefore poorer postural stability [5]. Values were analyzed continuously without transformation or categorization.

### 2.7. Mean COP Velocity

Mean COP velocity was a secondary posturographic outcome and was expressed in millimeters per second (mm/s) [15]. It represented the average rate of displacement of the COP trajectory over the complete 30-s recording interval and was derived from COP path displacement relative to trial duration [15]. The mean value from three valid trials was retained for each limb and condition. Higher mean COP velocity indicated greater postural corrective activity and poorer static postural control [15]. Identical acquisition duration and processing procedures were used across all limbs and experimental conditions.

### 2.8. Visual Condition and Visual-Dependence Response

Single-task postural control was assessed under eyes-open (EO) and eyes-closed (EC) conditions [16,17]. During EO trials, participants maintained visual fixation on a circular target positioned at approximately eye level 2 m directly in front of the force platform. During EC trials, participants assumed the identical unilateral stance position and closed their eyes immediately before initiation of data acquisition, maintaining eye closure throughout the 30-s recording [17]. Stance configuration, trial duration, instructions, and data-acquisition procedures were otherwise identical between visual conditions. COP path length, ellipse area, and mean COP velocity were obtained separately for each limb and condition. The response to visual deprivation was calculated at the participant level as EC minus EO for each posturographic outcome; positive values therefore represented increased sway following removal of visual input. Change scores were analyzed as continuous variables without application of a diagnostic cutoff [17].

### 2.9. Cognitive Dual-Task Condition

Cognitive dual-task testing was performed during unilateral eyes-open stance using the same stance configuration and 30-s acquisition period employed for the eyes-open single-task condition [16]. Concurrent cognitive loading consisted of continuous serial subtraction by threes from a randomly selected three-digit starting number between 200 and 300. Immediately before each dual-task trial, the examiner verbally provided the starting number, after which the participant subtracted three successively and stated each answer aloud throughout the trial. A different starting number was used for each trial to minimize learning and memorization effects. Participants were instructed to perform the postural and cognitive tasks concurrently without deliberately prioritizing either task. The examiner recorded responses but provided no feedback regarding accuracy during testing. Cognitive performance was monitored to confirm engagement with the secondary task; because the study outcome concerned cognitive interference with postural control, cognitive accuracy was not included as a primary or secondary outcome. COP path length, ellipse area, and mean COP velocity were recorded during the dual-task condition and compared with the corresponding eyes-open single-task condition for the same participant and limb. Absolute cognitive-task effects were calculated as dual-task minus single-task values, with positive change scores indicating increased postural sway during cognitive loading.

### 2.10. Dual-Task Cost in COP Path Length

COP path-length dual-task cost (DTC) was the principal derived cognitive-motor outcome and quantified the proportional deterioration in postural control associated with concurrent cognitive loading [18]. DTC was calculated separately for every participant and limb from participant-level values according to the equation: DTC (%) = [(dual-task COP path length − eyes-open single-task COP path length)/eyes-open single-task COP path length] × 100 [18]. A value of 0% indicated no task-related change, a positive value indicated increased COP path length during dual-tasking, and a negative value indicated decreased COP path length. DTC was treated as a continuous variable, and no diagnostic cutoff was applied. Reconstructed-limb DTC was compared with control reference-limb DTC and ACLR contralateral-limb DTC and was subsequently used as the dependent variable in the correlation and multiple linear regression analyses.

### 2.11. Limb Status and Interlimb Differences

In the ACLR group, the surgically treated limb was designated the reconstructed limb and the opposite limb the contralateral limb. In healthy controls, a reference limb was assigned before posturographic testing to reproduce the right-to-left distribution of reconstructed limbs in the ACLR group (19 right and 16 left), with the remaining limb designated the opposite limb. Reference-limb assignment was performed independently of posturographic performance and was not altered after testing. Limb dominance was recorded separately and was not used to redefine surgical or reference-limb status. Interlimb differences were examined by direct within-participant comparison of reconstructed versus contralateral limbs in the ACLR group and reference versus opposite limbs in healthy controls. No normalized limb-symmetry index was calculated; consequently, interlimb findings were expressed as direct differences in the original outcome units or, for DTC, in percentage points.

### 2.12. Postoperative and Return-to-Activity Duration

Four temporal variables were examined in relation to reconstructed-limb COP path-length DTC and were expressed in months [19]. Time since ACL reconstruction was calculated from the operative date to the posturographic assessment date. Time since return to unrestricted sport/activity was calculated from the documented date on which the participant was medically cleared to resume sport or habitual physical activity without postoperative restrictions to the assessment date. Supervised rehabilitation duration was defined as the interval from initiation to discharge from structured postoperative rehabilitation supervised by a physiotherapist. Time from ACL injury to reconstruction was calculated from the documented injury date to the operative date. Operative and return-to-activity dates were preferentially obtained from surgical, orthopedic, or rehabilitation records and cross-checked during the participant interview. All variables were retained as continuous measures. The four temporal variables were examined in bivariate analyses, while time since ACL reconstruction and time since return to unrestricted sport/activity were the prespecified temporal predictors entered into the multivariable model because they directly addressed the postoperative and return-to-activity components of the study objective.

### 2.13. Demographic, Anthropometric, and Activity Variables

Age and sex were recorded during the baseline interview and verified against the clinical record where available. Standing height was measured without shoes to the nearest 0.1 cm using a wall-mounted stadiometer (Seca 206, Seca GmbH & Co. KG, Hamburg, Germany), and body mass was measured in light clothing without shoes to the nearest 0.1 kg using a calibrated digital scale. BMI was calculated as body mass in kilograms divided by height in meters squared (kg/m^2^). Limb dominance was defined as the limb the participant would preferentially use to kick a ball and was recorded as right or left. Habitual weekly sport/training exposure was determined by structured interview as the average number of hours per week spent in organized sport, resistance training, running, or other planned moderate-to-vigorous physical training during the preceding four weeks. For the purposes of this study, physical activity was characterized by self-reported participation in organized sport or planned moderate-to-vigorous physical training, with habitual exposure quantified as average weekly training hours during the preceding four weeks. This measure included activities such as organized sport, resistance training, and running but did not distinguish team-based from individual sporting participation or provide separate estimates of activity intensity across exercise modalities. Consequently, weekly training exposure was used to characterize habitual physical activity and facilitate group-level matching rather than to establish sport-specific demands, competitive participation, or equivalence of exercise intensity between groups. These characteristics were used to describe the sample and achieve group-level frequency matching rather than as primary explanatory variables.

### 2.14. ACLR-Specific Clinical Variables

Operated side, ACL graft type, and concomitant meniscal procedure were obtained from operative or orthopedic records and categorized as reported in Table 1. Postoperative knee range of motion was assessed at baseline using a standard 30-cm universal goniometer (Baseline, Fabrication Enterprises Inc., White Plains, NY, USA). Participants were positioned supine, with the greater trochanter, lateral femoral epicondyle, and lateral malleolus used as anatomical landmarks. Active knee flexion was measured from full available extension to maximal voluntary flexion and recorded to the nearest degree. Knee extension deficit was defined as the number of degrees short of neutral 0° extension. Two measurements were obtained by the same trained assessor, and their mean was retained for analysis. These clinical characteristics were used to describe the ACLR cohort and were not included as primary predictors in the posturographic analyses.

### 2.15. Data Analysis

All statistical analyses were performed using IBM SPSS Statistics, version 24.0 (IBM Corp., Armonk, NY, USA). Continuous variables were summarized as mean ± standard deviation (SD), whereas categorical variables were presented as frequencies and percentages. Distributional assumptions for continuous variables were assessed using the Shapiro–Wilk test together with visual inspection of histograms and normal Q–Q plots; no material departures from normality were identified, and parametric procedures were therefore applied. Homogeneity of variance for independent-group comparisons was evaluated using Levene’s test. Baseline continuous characteristics were compared between the ACLR and healthy-control groups using independent-samples *t*-tests. For the posturographic outcomes—center-of-pressure (COP) path length, ellipse area, and mean COP velocity—independent-samples *t*-tests compared the ACLR reconstructed limb with the control reference limb under eyes-open, eyes-closed, and cognitive dual-task conditions and compared the corresponding participant-level eyes-closed minus eyes-open and dual-task minus single-task change scores. The eyes-open condition served as the single-task reference for cognitive dual-task analyses. COP path-length dual-task cost (DTC) was calculated separately for each participant and limb as [(dual-task COP path length − eyes-open single-task COP path length)/eyes-open single-task COP path length] × 100. Between-group DTC comparisons were performed using independent-samples *t*-tests, whereas prespecified within-group limb comparisons (reconstructed versus contralateral limb in the ACLR group and reference versus opposite limb in controls) were performed using paired-samples *t*-tests. Cohen’s d was reported as the standardized effect size for between-group DTC comparisons, and mean differences were presented with 95% confidence intervals (CIs). Within the ACLR group, Pearson correlation coefficients (r) were used to examine associations between reconstructed-limb DTC and time since ACL reconstruction, time since return to unrestricted sport/activity, supervised rehabilitation duration, and time from ACL injury to reconstruction; 95% CIs for r were derived using Fisher’s r-to-z transformation. Multiple linear regression was subsequently performed with reconstructed-limb DTC as the dependent variable and time since ACL reconstruction and time since return to unrestricted sport/activity as the two prespecified predictors, corresponding directly to the temporal factors addressed by the study objective. Prior to regression analysis, linearity was evaluated using scatterplots, residual normality using Q–Q plots, homoscedasticity using standardized residual-versus-predicted-value plots, multicollinearity using tolerance and variance inflation factor statistics, and potentially influential observations using standardized residuals and Cook’s distance; no material violations requiring transformation or exclusion were identified. Standardized regression coefficients (β), 95% CIs, R^2^, adjusted R^2^, and the overall F statistic were reported. Confidence intervals for standardized regression coefficients were estimated using nonparametric bootstrapping with 5000 resamples and bias-corrected 95% confidence intervals. All statistical tests were two-sided, and *p* < 0.05 was considered statistically significant. To control the family-wise error rate arising from multiple hypothesis tests, the Holm–Bonferroni step-down procedure was applied within predefined families of related comparisons. The four bivariate correlations between reconstructed-limb DTC and postoperative temporal variables were treated as one family, whereas the two prespecified temporal predictors in the multiple linear regression model were treated as a separate family. Adjusted *p*-values were calculated by ordering the unadjusted *p*-values from smallest to largest, multiplying each by the number of remaining hypotheses, and enforcing monotonicity across successive comparisons. Statistical significance was determined using an adjusted *p*-value threshold of 0.05. Unadjusted and adjusted *p*-values were distinguished in reporting to facilitate interpretation of findings potentially affected by multiple testing.

## 3. Results

The ACLR and healthy control groups were comparable in demographic, anthropometric, and activity-related characteristics, with no significant between-group differences in age, height, body mass, BMI, or weekly sport/training exposure (all *p* > 0.05) (Table 1). Participants in the ACLR group were assessed 15.80 ± 4.30 months after reconstruction and 7.10 ± 3.00 months after return to unrestricted sport/activity, following 8.60 ± 1.90 months of supervised rehabilitation. Hamstring tendon autograft was the most frequently used graft (62.86%), while 40.00% of participants underwent a concomitant meniscal procedure. Postoperative knee flexion was 137.20 ± 5.80°, with a small residual extension deficit of 0.70 ± 1.00°.

Single-task postural control was consistently poorer in the reconstructed ACLR limb than in the control reference limb, with between-group differences becoming more pronounced when visual input was removed (Table 2). With eyes closed, COP path length was 720.00 ± 118.00 mm in the reconstructed limb compared with 552.00 ± 92.00 mm in the control reference limb (mean difference, 168.00 mm; 95% CI, 117.48–218.52; *p* < 0.001), while ellipse area differed by 183.00 mm^2^ (95% CI, 121.10–244.90; *p* < 0.001). Mean COP velocity was also higher in the reconstructed limb under eyes-closed conditions (24.00 ± 3.93 vs. 18.40 ± 3.07 mm/s; *p* < 0.001), and removal of vision produced a substantially greater increase in COP path length in the reconstructed limb than in controls (250.00 ± 89.83 vs. 150.00 ± 70.39 mm; *p* < 0.001).

Cognitive dual-tasking accentuated postural-control impairment in the reconstructed ACLR limb compared with the control reference limb across the principal posturographic outcomes (Table 3). During dual-tasking, COP path length was 552.00 ± 96.00 mm in the reconstructed limb versus 430.00 ± 73.00 mm in controls (mean difference, 122.00 mm; 95% CI, 81.27–162.73; *p* < 0.001), with a significantly greater task-related increase of 82.00 ± 67.86 versus 28.00 ± 52.96 mm (mean difference, 54.00 mm; *p* < 0.001). Dual-task deterioration was also greater for ellipse area (mean change difference, 45.00 mm^2^; *p* = 0.015) and mean COP velocity (mean change difference, 1.80 mm/s; *p* < 0.001).

Dual-task cost for COP path length was substantially greater in the reconstructed ACLR limb than in the control reference limb (17.45 ± 10.20% vs. 6.97 ± 6.80%; mean difference, 10.48 percentage points; 95% CI, 6.34–14.63; Cohen’s d = 1.21; *p* < 0.001) (Table 4). A smaller but significant difference was also present between the contralateral ACLR and opposite control limbs (11.00 ± 8.40% vs. 6.78 ± 6.60%; *p* = 0.023). Within the ACLR group, dual-task cost was significantly greater in the reconstructed than the contralateral limb (mean difference, 6.45 percentage points; *p* < 0.001), whereas no meaningful interlimb difference was evident among controls (*p* = 0.880).

**Figure 2:**
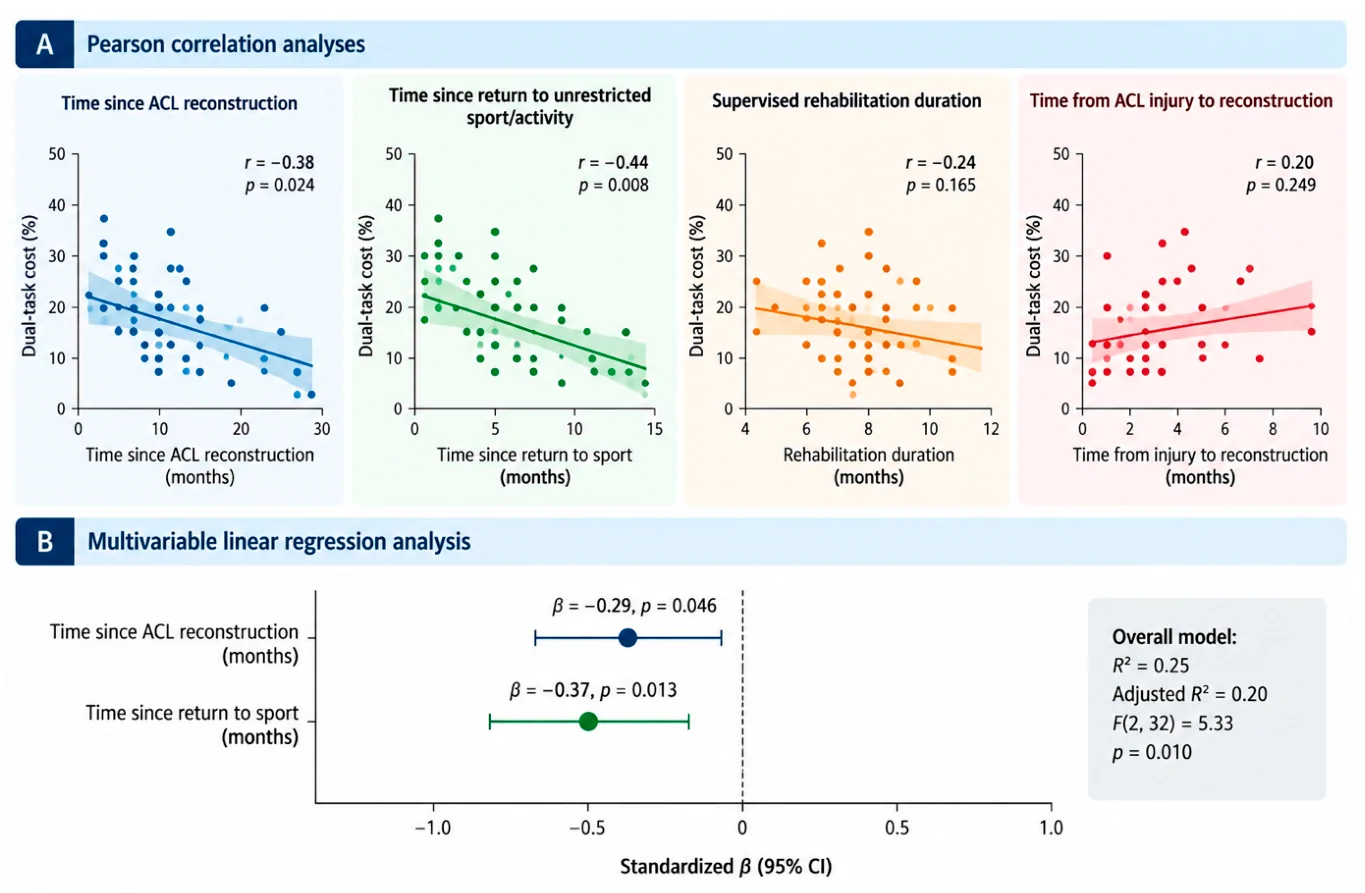
Associations between postoperative temporal variables and reconstructed-limb center-of-pressure path-length dual-task cost (DTC) in participants following anterior cruciate ligament reconstruction (ACLR; *n* = 35). (**A**) Pearson correlation analyses illustrating the relationships of DTC (%) with time since ACL reconstruction (months) and time since medical clearance for unrestricted sport/activity (months). (**B**) Multiple linear regression analysis examining the independent associations of both temporal predictors with reconstructed-limb DTC. Pearson correlation coefficients (r), standardized regression coefficients (β), and corresponding *p*-values are reported in Table 5. DTC is expressed as a percentage, and temporal variables are expressed in months.

In the unadjusted Pearson correlation analyses, longer time since ACL reconstruction (r = −0.38; *p* = 0.024) and longer time since return to unrestricted sport/activity (r = −0.44; *p* = 0.008) were associated with lower reconstructed-limb DTC (Table 5 and Figure 2). Following Holm–Bonferroni correction across the four temporal correlations, the association with time since return to unrestricted activity remained statistically significant (adjusted *p* = 0.032), whereas the association with time since reconstruction did not (adjusted *p* = 0.072). Supervised rehabilitation duration and time from ACL injury to reconstruction were not significantly associated with DTC (adjusted *p* = 0.330 for both). In the multiple linear regression model, time since reconstruction (standardized β = −0.29; unadjusted *p* = 0.046; adjusted *p* = 0.046) and time since return to unrestricted sport/activity (β = −0.37; unadjusted *p* = 0.013; adjusted *p* = 0.026) retained statistically significant inverse associations with DTC after correction across the two prespecified regression coefficients. The overall model explained 25% of the variance in reconstructed-limb DTC (R^2^ = 0.25; adjusted R^2^ = 0.20; F [2,32] = 5.33; *p* = 0.010).

## 4. Discussion

The present study aimed to characterize persistent sensorimotor alterations following anterior cruciate ligament reconstruction by examining single-task postural control under different visual conditions, determining the influence of cognitive dual-tasking on postural performance, and exploring whether postoperative and return-to-activity duration were associated with residual dual-task deficits. The findings demonstrated that individuals following ACL reconstruction exhibited poorer unilateral postural control than healthy controls, with deficits being particularly evident in the reconstructed limb and becoming more pronounced when visual input was removed. Cognitive dual-tasking further accentuated these impairments, producing greater deterioration in center-of-pressure measures and a higher dual-task cost in the reconstructed limb, while the contralateral limb showed comparatively smaller deficits. In contrast, interlimb differences among healthy controls were minimal. Furthermore, longer time since reconstruction and longer time since return to unrestricted sport or activity were associated with lower dual-task cost, and both temporal factors retained independent associations in the multivariable analysis. Collectively, these findings suggest that residual postural-control impairments after ACL reconstruction are sensitive to both visual deprivation and cognitive loading and may persist despite clearance for unrestricted sport or activity.

The greater postural sway observed in the reconstructed ACLR limb, particularly under eyes-closed conditions, indicates that residual sensorimotor deficits remain detectable after completion of rehabilitation and return to unrestricted activity [20]. Two mechanisms provide a plausible explanation for this pattern. First, ACL injury and reconstruction alter the proprioceptive contribution of mechanoreceptors associated with the native ligament, potentially reducing the quality of afferent information available for sensorimotor regulation of knee position and postural stability [21]. This interpretation is consistent with Henriksson et al. [22], who reported altered postural control following ACL reconstruction and emphasized the relevance of proprioceptive recovery during functional rehabilitation. Second, removal of visual information increases reliance on somatosensory and proprioceptive inputs; consequently, deficits that may be partially compensated for when vision is available can become more evident during eyes-closed stance [22,23]. Consistent with this explanation, Stańczak et al. [3] demonstrated impaired postural control in individuals with ACL insufficiency and related these alterations to reduced proprioceptive afferent input. Furthermore, the systematic review by Gholami et al. [8] concluded that postural-control abnormalities may persist following ACL reconstruction despite surgical and rehabilitative management, supporting the present finding that conventional clinical recovery does not necessarily indicate complete restoration of sensorimotor control. Collectively, the greater COP path length, ellipse area, and COP velocity in the reconstructed limb, together with their amplification following visual deprivation, support the presence of persistent sensory-dependent postural-control impairment after ACL reconstruction [8].

The disproportionate deterioration in postural control during cognitive dual-tasking, particularly in the reconstructed limb, indicates that residual ACLR-related sensorimotor impairment becomes more apparent when attentional resources must simultaneously support postural regulation and a secondary cognitive task [24]. Two factors may account for this response. First, when automatic sensorimotor regulation is compromised after ACL injury and reconstruction, maintenance of postural stability may require greater attentional involvement; introducing a concurrent cognitive task can therefore reduce the attentional resources available for compensatory postural regulation [25]. Sharouf et al. [9] similarly demonstrated that dual-tasking modifies postural control in individuals with unilateral ACL injury, supporting an interaction between cognitive demand and ACL-related sensorimotor dysfunction. More recent work examining individuals after ACL reconstruction has likewise reported alterations in proprioception and related sensorimotor performance under cognitive dual-task conditions, reinforcing the relevance of cognitive loading when evaluating postoperative function [26]. Second, the inverse relationships between dual-task cost and both time since reconstruction and time since return to unrestricted activity are consistent with progressive improvement in the efficiency of sensorimotor control with continued recovery and exposure to complex functional environments, although the cross-sectional design does not establish temporal causality [27]. Cross-sectional research comparing ACL-reconstructed individuals with healthy controls under concurrent cognitive tasks has also demonstrated that secondary cognitive loading can reveal postural-control characteristics that are less apparent during conventional single-task testing. Taken together, the elevated dual-task cost in the reconstructed limb, the smaller alteration in the contralateral limb, and the absence of a meaningful interlimb difference in controls indicate that cognitive-motor testing may provide complementary information about residual postural-control deficits that is not fully captured by single-task assessment alone [28].

The contralateral-limb findings warrant further consideration because dual-task cost was significantly greater in the non-reconstructed limb of ACLR participants than in the corresponding limb of healthy controls (11.00 ± 8.40% versus 6.78 ± 6.60%; *p* = 0.023). Although the reconstructed limb exhibited the greatest dual-task interference, this additional between-group difference suggests that altered cognitive-motor postural performance may extend beyond the surgically treated knee. One possible explanation involves bilateral sensorimotor adaptations following unilateral ACL injury, including changes in central sensory processing, motor planning, and attentional regulation [29]. Altered movement strategies, compensatory loading, and reduced exposure to demanding physical activities during rehabilitation may also contribute. Psychological factors, including fear of reinjury and reduced movement confidence, represent further plausible influences on motor performance [30]. However, these mechanisms were not directly investigated, and the observed contralateral-limb difference cannot establish central neural reorganization or a psychological effect of injury [30]. Although contralateral ACL injury is a recognized concern following return to sport, the present cross-sectional findings do not establish whether greater contralateral-limb dual-task cost is associated with subsequent injury risk. Future longitudinal studies incorporating bilateral postural assessment, psychological measures, neurophysiological evaluation, and prospective injury surveillance are needed to clarify the mechanisms and clinical significance of these observations.

### 4.1. Clinical Significance

The present findings have direct clinical relevance for the assessment of sensorimotor recovery after ACL reconstruction because they demonstrate that residual postural-control deficits may remain detectable despite medical clearance for unrestricted sport or physical activity. Specifically, reconstructed-limb impairments became more evident when visual input was removed and when a concurrent cognitive task was imposed, while dual-task cost showed a clear reconstructed-to-contralateral limb difference that was not observed between limbs in healthy controls. These findings indicate that conventional single-task testing under visually available conditions may not fully characterize residual postural-control impairment. Incorporating unilateral force-platform assessment under eyes-closed and cognitive dual-task conditions may therefore provide clinically relevant complementary information when evaluating post-ACLR sensorimotor function. The observed inverse associations between reconstructed-limb dual-task cost and both time since reconstruction and time since return to unrestricted activity further indicate that the postoperative and return-to-activity timeline should be considered when interpreting dual-task postural performance. Accordingly, Technobody Isofree assessment of COP path length and its dual-task cost may complement existing clinical and functional measures by identifying residual sensorimotor asymmetry under conditions that place greater demands on sensory integration and cognitive-motor control.

The present findings demonstrate measurable differences in static unilateral postural control between individuals following ACL reconstruction and matched healthy controls, particularly during visual deprivation and cognitive dual-tasking. These observations suggest that force-platform posturography may provide complementary information about postural performance under controlled sensory and attentional conditions [30]. However, the observed differences should not be interpreted as established indicators of clinically meaningful functional impairment or readiness for unrestricted sporting participation. The study did not determine diagnostic thresholds, evaluate prospective injury outcomes, or establish the predictive validity of center-of-pressure measures or dual-task cost for subsequent knee injury or reinjury. Moreover, static unilateral stance does not reproduce the dynamic neuromuscular, perceptual, and decision-making demands of sport-specific activities such as cutting, landing, acceleration, and rapid changes in direction [31]. The inverse associations between dual-task cost and postoperative temporal variables should likewise be interpreted as cross-sectional relationships rather than evidence of longitudinal recovery. Accordingly, force-platform assessment under visual deprivation and cognitive loading should be regarded as an exploratory adjunct to established clinical and functional evaluations, rather than an independent criterion for return-to-sport decisions [31]. Prospective investigations are required to establish the clinical relevance, responsiveness, and prognostic value of these measures before their routine use in return-to-sport decision-making can be recommended.

### 4.2. Limitations of the Study and Areas for Future Research

Several limitations should be considered when interpreting these findings. First, the cross-sectional design precludes causal inference and does not permit assessment of individual recovery trajectories or changes in postural control over time. Accordingly, the observed associations between postoperative duration, time since medical clearance for unrestricted activity, and dual-task cost should not be interpreted as evidence of progressive recovery. Second, the relatively small sample of 35 ACLR participants and 35 matched controls, together with the restricted age range of 18–35 years and inclusion of physically active individuals who had completed rehabilitation, limits the generalizability of the findings to older adults, less active populations, patients undergoing revision reconstruction, individuals with bilateral ACL injuries, and those at earlier rehabilitation stages. Furthermore, return to unrestricted activity was defined by documented medical clearance rather than objectively verified restoration of preinjury sporting participation or performance. Therefore, the temporal associations reflect time elapsed since clearance and cannot establish whether resumption of previous sporting levels influenced postural-control outcomes. Third, although the groups were matched for major demographic and activity-related characteristics, potential clinical confounding remains. Graft type, concomitant meniscal procedures, rehabilitation exposure, and postoperative duration may influence postural-control performance. These characteristics were documented but were not included as covariates in the principal regression analyses, preventing determination of their independent contributions. The limited sample size and unequal distribution of surgical characteristics also restricted meaningful subgroup analyses. Fourth, the assessment was confined to static unilateral stance using selected center-of-pressure outcomes, which may not adequately represent the neuromuscular demands of dynamic sporting activities, including landing, cutting, and rapid directional changes. Similarly, the serial-subtraction dual-task paradigm provided a standardized cognitive challenge but did not reproduce the complex perceptual and decision-making demands encountered during sport. Habitual physical activity was characterized primarily by self-reported weekly training exposure without detailed classification of sporting discipline, competitive level, or exercise intensity, limiting assessment of whether sport-specific demands influenced postural-control performance. Additionally, unsuccessful posturographic trials were repeated according to standardized validity criteria, but their frequency was not analyzed as an independent outcome. Consequently, the potential relationship between trial failure, functional balance capacity, and sport-specific performance remains undetermined. Furthermore, the study did not assess the diagnostic accuracy, clinically meaningful thresholds, or prospective predictive validity of the posturographic outcomes. No subsequent knee injury or reinjury outcomes were recorded, preventing conclusions regarding the ability of center-of-pressure measures or dual-task cost to identify individuals at increased injury risk. Consequently, the observed between-group differences and interlimb asymmetries should be interpreted as laboratory-based measures of postural performance rather than validated indicators of sporting readiness or future clinical outcomes. Finally, the relatively large number of statistical comparisons increases the potential for type I error, particularly for associations approaching the conventional significance threshold, and warrants cautious interpretation of exploratory findings. Future prospective, multicenter studies involving larger and more heterogeneous cohorts should incorporate objectively verified return-to-sport participation and performance, account for graft selection, meniscal treatment, and rehabilitation characteristics, and evaluate postural-control changes longitudinally. Additional research should establish clinically meaningful thresholds for posturographic asymmetry and dual-task cost and investigate their relationships with dynamic movement performance, subsequent knee injury, and longer-term functional outcomes.

## 5. Conclusions

Individuals who had returned to unrestricted sport or physical activity following primary unilateral ACL reconstruction demonstrated persistent alterations in unilateral postural control compared with matched healthy controls. These deficits were most apparent in the reconstructed limb and were accentuated by visual deprivation and concurrent cognitive loading, as reflected by greater COP path length, ellipse area, mean COP velocity, and reconstructed-limb dual-task cost. The reconstructed limb also demonstrated greater dual-task cost than the contralateral limb, whereas comparable interlimb differences were not evident in healthy controls. In addition, longer time since ACL reconstruction and longer time since return to unrestricted sport or activity were independently associated with lower reconstructed-limb dual-task cost. These findings demonstrate that eyes-closed and cognitive dual-task posturography using the Technobody Isofree platform can identify residual sensorimotor asymmetry after ACL reconstruction that remains evident following clearance for unrestricted activity.

## Figures and Tables

**Table 1:** Demographic, anthropometric, activity-related, and clinical characteristics of participants following unilateral primary anterior cruciate ligament reconstruction and matched healthy controls.

	ACLR Group (*n* = 35)	Healthy Controls (*n* = 35)	Mean Difference (ACLR − Control)	95% CI of Difference	*p*-Value
Age, years	27.80 ± 5.10	27.40 ± 5.00	0.40	−2.01 to 2.81	0.741
Height, cm	174.20 ± 8.40	173.70 ± 8.10	0.50	−3.44 to 4.44	0.801
Body mass, kg	75.40 ± 10.70	73.90 ± 10.10	1.50	−3.46 to 6.46	0.548
BMI, kg/m^2^	24.80 ± 2.90	24.40 ± 2.70	0.40	−0.94 to 1.74	0.552
Weekly sport/training exposure, h/week	5.30 ± 2.10	5.50 ± 2.00	-0.20	−1.18 to 0.78	0.685
Sex, *n* (%)					
Male	24 (68.57)	24 (68.57)	—	—	—
Female	11 (31.43)	11 (31.43)	—	—	—
Dominant limb, *n* (%)					
Right	31 (88.57)	31 (88.57)	—	—	—
Left	4 (11.43)	4 (11.43)	—	—	—
Operated limb, *n* (%)	Right: 19 (54.29) Left: 16 (45.71)	—	—	—	—
Time from ACL injury to reconstruction, months	2.90 ± 2.10	—	—	—	—
Time since ACL reconstruction, months	15.80 ± 4.30	—	—	—	—
Time since return to unrestricted sport/activity, months	7.10 ± 3.00	—	—	—	—
Supervised rehabilitation duration, months	8.60 ± 1.90	—	—	—	—
Graft type, *n* (%)					
Hamstring tendon autograft	22 (62.86)	—	—	—	—
Bone–patellar tendon–bone autograft	10 (28.57)	—	—	—	—
Quadriceps tendon autograft	3 (8.57)	—	—	—	—
Concomitant meniscal procedure, *n* (%)					
None	21 (60.00)	—	—	—	—
Meniscal repair	9 (25.71)	—	—	—	—
Partial meniscectomy	5 (14.29)	—	—	—	—
Postoperative knee flexion, degrees	137.20 ± 5.80	—	—	—	—
Knee extension deficit, degrees	0.70 ± 1.00	—	—	—	—

Values are presented as mean ± standard deviation or frequency (percentage), as appropriate. Between-group mean differences and 95% confidence intervals refer to comparisons between the ACLR and healthy-control groups. ACLR-specific clinical characteristics are presented descriptively. Abbreviations: ACL, anterior cruciate ligament; ACLR, anterior cruciate ligament reconstruction; BMI, body mass index; CI, confidence interval.

**Table 2:** Single-task postural-control outcomes during unilateral stance under eyes-open and eyes-closed conditions in participants following anterior cruciate ligament reconstruction and matched healthy controls.

Outcome/Visual Condition	ACLR Reconstructed Limb (*n* = 35)	ACLR Contralateral Limb (*n* = 35)	Control Reference Limb (*n* = 35)	Control Opposite Limb (*n* = 35)	Between-Group Mean Difference	95% CI	*p*-Value
Center-of-pressure path length, mm							
Eyes open	470.00 ± 82.00	418.00 ± 72.00	402.00 ± 68.00	398.00 ± 66.00	68.00	32.05 to 103.95	<0.001
Eyes closed	720.00 ± 118.00	602.00 ± 101.00	552.00 ± 92.00	545.00 ± 90.00	168.00	117.48 to 218.52	<0.001
Eyes-closed − eyes-open change	250.00 ± 89.83	184.00 ± 77.02	150.00 ± 70.39	147.00 ± 68.80	100.00	61.47 to 138.53	<0.001
Ellipse area, mm^2^							
Eyes open	342.00 ± 98.00	285.00 ± 84.00	262.00 ± 77.00	258.00 ± 75.00	80.00	37.92 to 122.08	<0.001
Eyes closed	565.00 ± 148.00	438.00 ± 126.00	382.00 ± 108.00	376.00 ± 105.00	183.00	121.10 to 244.90	<0.001
Eyes-closed − eyes-open change	223.00 ± 118.76	153.00 ± 101.15	120.00 ± 87.26	118.00 ± 84.85	103.00	53.21 to 152.79	<0.001
Mean COP velocity, mm/s							
Eyes open	15.67 ± 2.73	13.93 ± 2.40	13.40 ± 2.27	13.27 ± 2.20	2.27	1.07 to 3.47	<0.001
Eyes closed	24.00 ± 3.93	20.07 ± 3.37	18.40 ± 3.07	18.17 ± 3.00	5.60	3.92 to 7.28	<0.001
Eyes-closed − eyes-open change	8.33 ± 2.99	6.14 ± 2.57	5.00 ± 2.35	4.90 ± 2.29	3.33	2.05 to 4.61	<0.001

Abbreviations: ACLR, anterior cruciate ligament reconstruction; CI, confidence interval; COP, center of pressure.

**Table 3:** Effect of cognitive dual-tasking on unilateral postural control in participants following anterior cruciate ligament reconstruction and matched healthy controls.

Outcome/Task Condition	ACLR Reconstructed Limb (*n* = 35)	ACLR Contralateral Limb (*n* = 35)	Control Reference Limb (*n* = 35)	Control Opposite Limb (*n* = 35)	Between-Group Mean Difference	95% CI	*p*-Value
Center-of-pressure path length, mm							
Single task	470.00 ± 82.00	418.00 ± 72.00	402.00 ± 68.00	398.00 ± 66.00	68.00	32.05 to 103.95	<0.001
Dual task	552.00 ± 96.00	464.00 ± 80.00	430.00 ± 73.00	425.00 ± 71.00	122.00	81.27 to 162.73	<0.001
Dual-task − single-task change	82.00 ± 67.86	46.00 ± 57.36	28.00 ± 52.96	27.00 ± 51.47	54.00	24.94 to 83.06	<0.001
Ellipse area, mm^2^							
Single task	342.00 ± 98.00	285.00 ± 84.00	262.00 ± 77.00	258.00 ± 75.00	80.00	37.92 to 122.08	<0.001
Dual task	411.00 ± 113.00	324.00 ± 91.00	286.00 ± 82.00	281.00 ± 80.00	125.00	77.83 to 172.17	<0.001
Dual-task − single-task change	69.00 ± 85.51	39.00 ± 70.29	24.00 ± 63.76	23.00 ± 62.17	45.00	8.97 to 81.03	0.015
Mean COP velocity, mm/s							
Single task	15.67 ± 2.73	13.93 ± 2.40	13.40 ± 2.27	13.27 ± 2.20	2.27	1.07 to 3.47	<0.001
Dual task	18.40 ± 3.20	15.47 ± 2.67	14.33 ± 2.43	14.17 ± 2.37	4.07	2.71 to 5.43	<0.001
Dual-task − single-task change	2.73 ± 2.26	1.54 ± 1.91	0.93 ± 1.76	0.90 ± 1.72	1.80	0.83 to 2.77	<0.001

Abbreviations: ACLR, anterior cruciate ligament reconstruction; CI, confidence interval; COP, center of pressure.

**Table 4:** Dual-task cost in center-of-pressure path length during unilateral stance in participants following anterior cruciate ligament reconstruction and matched healthy controls.

Comparison	DTC, Mean ± SD, %	Comparator DTC, Mean ± SD, %	Mean Difference, Percentage Points	95% CI	Effect Size, Cohen’s d	*p*-Value
Between-group comparisons						
Reconstructed ACLR limb vs. control reference limb	17.45 ± 10.20	6.97 ± 6.80	10.48	6.34 to 14.63	1.21	<0.001
Contralateral ACLR limb vs. opposite control limb	11.00 ± 8.40	6.78 ± 6.60	4.22	0.61 to 7.83	0.56	0.023
Within-group limb comparisons						
ACLR: reconstructed vs contralateral limb	17.45 ± 10.20	11.00 ± 8.40	6.45	3.06 to 9.84	—	<0.001
Controls: reference vs opposite limb	6.97 ± 6.80	6.78 ± 6.60	0.19	−2.23 to 2.60	—	0.880

Data are presented as mean ± standard deviation. Dual-task cost was calculated for each participant as [(dual-task COP path length − single-task COP path length)/single-task COP path length] × 100. Abbreviations: ACLR, anterior cruciate ligament reconstruction; CI, confidence interval; COP, center of pressure; DTC, dual-task cost; SD, standard deviation.

**Table 5:** Associations of postoperative and return-to-activity duration with reconstructed-limb dual-task postural-control deficit following anterior cruciate ligament reconstruction.

Analysis/Predictor	Predictor Value, Mean ± SD	r/Standardized β	95% CI	*p*-Value	Holm-Adjusted *p*-Value
Pearson correlation analyses					
Time since ACL reconstruction, months	15.80 ± 4.30	−0.38	−0.63 to −0.05	0.024	0.072
Time since return to unrestricted sport/activity, months	7.10 ± 3.00	−0.44	−0.67 to −0.13	0.008	0.032
Supervised rehabilitation duration, months	8.60 ± 1.90	−0.24	−0.53 to 0.10	0.165	0.330
Time from ACL injury to reconstruction, months	2.90 ± 2.10	0.20	−0.14 to 0.50	0.249	0.330
Multiple linear regression: reconstructed-limb DTC (%) as dependent variable					
Time since ACL reconstruction, months	15.80 ± 4.30	−0.29	−0.58 to −0.01	0.046	0.046
Time since return to unrestricted sport/activity, months	7.10 ± 3.00	−0.37	−0.66 to −0.08	0.013	0.026
Overall regression model	—	R^2^ = 0.25; adjusted R^2^ = 0.20	F(2, 32) = 5.33	0.010	

Data are presented for the ACLR group (*n* = 35). The dependent variable was reconstructed-limb COP path-length DTC. Pearson’s r with 95% confidence intervals is reported for bivariate associations. Standardized regression coefficients (β) are reported for the multiple linear regression model. Holm-adjusted *p*-values account for multiple testing within the four temporal correlation analyses and, separately, within the two prespecified regression coefficients. Statistical significance after correction was defined as adjusted *p* < 0.05. The overall regression model *p*-value is reported without adjustment as an omnibus test. Abbreviations: ACL, anterior cruciate ligament; ACLR, anterior cruciate ligament reconstruction; β, standardized regression coefficient; CI, confidence interval; COP, center of pressure; DTC, dual-task cost; SD, standard deviation.

## Data Availability

The dataset supporting the findings of this study is publicly available in the Zenodo repository at https://doi.org/10.5281/zenodo.23313883. This DOI represents all versions of the dataset and resolves to the latest available version. The initial dataset (Version 1), published on 12 October 2026, is accessible at https://doi.org/10.5281/zenodo.23313884.

## Abbreviations

The following abbreviations are used in this manuscript:

ACL	Anterior Cruciate Ligament
ACLR	Anterior Cruciate Ligament Reconstruction
BMI	Body Mass Index
COP	Center of Pressure
DTC	Dual-Task Cost
EC	Eyes Closed
EO	Eyes Open
SPSS	Statistical Package for the Social Sciences
